# Education and Examination

**Published:** 1917-12-29

**Authors:** 


### EDUCATION AND EXAMINATION.

Popular opinion places a high value on examinations as a guarantee of the efficiency of the individual who has successfully survived the ordeal. The judgment goes still further, and extends its verdict to the teacher and also to the educational system under which the candidate has been trained. With good examination results the method of training, it is concluded, must be good, and the test is accepted as final. Such, however, is by no means the view which prevails among educational experts, when, as is commonly the case, the teaching is undertaken by one authority and the examining by another. Inevitably, in such circumstances, the teacher is largely controlled by the demands of the examining board, for it is here that his worth would seem to be appraised. Results in the shape of successful candidates are necessary to him, and he must take the way most likely to attain them, whether this way provides or does not provide the educational discipline best fitted for the mental development of his pupils. To a growing extent teachers have resented this position, and have claimed that the examination should be dominated by the teaching, and not the teaching by the examination. They urge, that is, that they ought themselves to be free to decide and select the curriculum, and that the examination should be adjusted to the scheme of training which has been actually put into practice. The most direct way of granting this demand would be to arrange that the teacher and the examiner should be one and the same person. But to this plan there is the objection that by it the teacher is made the censor of his own work, and that his judgment must needs be a partial one. It is somewhere between the two extremes here indicated that is to be found the point where the teacher can exercise a reasonable influence on examinations without at the same time becoming a complete master of the situation.

An attempt is about to be made to reach this point in connection with the English system of secondary schools. At present these schools depend for an examination verdict on the examinations of outside bodies, such as the universities and the professions, which prescribe certain requirements as entrance tests. The teacher must therefore adjust his ambitions to these tests. The new arrangement includes a "Secondary Schools Examination Council," on which not only the universities and the managers of the schools will be represented, but also the teachers. Under this authority examinations will be held and a "leaving certificate" granted; and, what is obviously of great importance to the issue here discussed, each individual school may be examined on its own syllabus. There will thus be established definite standards of examination, upon which the teacher will exercise his share of influence, and the standard will be graded according as the pupil is obliged to leave school at sixteen or at eighteen years of age. An element in the success of the enterprise rests, among others, with the General Medical Council. The Council and kindred professional bodies may, and doubtless will, accept the "leaving certificate" as a sufficient test of preliminary education. But their full influence will only be exercised if they refuse to accept the certificates of outside examining bodies which are not recognised by the Secondary Schools Examination Council. Some of these may be obtained by a shorter process of education than that required by the new "leaving certificate," which, demanding a certain term of school attendance, pays recognition to the principle that teaching, and not examination, is the vital element in any sound educational scheme.

